# Social Jetlag Is Associated With Impaired Metabolic Control During a 1-Year Follow-Up

**DOI:** 10.3389/fphys.2021.702769

**Published:** 2021-09-02

**Authors:** Maria Carliana Mota, Catarina Mendes Silva, Laura Cristina Tibiletti Balieiro, Walid Makin Fahmy, Elaine Cristina Marqueze, Claudia Roberta de Castro Moreno, Cibele Aparecida Crispim

**Affiliations:** ^1^Faculty of Medicine of the Federal University of Uberlândia, Uberlândia, Brazil; ^2^Hospital and Maternity Municipal of Uberlândia, Uberlândia, Brazil; ^3^Public Health Graduate Program, Department of Epidemiology, Catholic University of Santos, Santos, Brazil; ^4^Department of Health, Life Cycles and Society, School of Public Health, University of São Paulo, São Paulo, Brazil; ^5^Stress Research Institute, Department of Psychology, Stockholm University, Stockholm, Sweden

**Keywords:** chronic diseases, metabolic parameters, sleep, circadian rhythms, social jetlag

## Abstract

Previous studies have identified social jetlag (SJL) as a risk factor for non-communicable chronic diseases (NCCDs), but its association with metabolic control over time is unclear in the literature. Therefore, we examined the influence of SJL on metabolic parameters and blood pressure (BP) in patients with NCCDs over a 1-year follow-up. This retrospective, longitudinal study included 625 individuals (age: 56.0 +12.0 years; 76% female) with NCCDs [type 2 diabetes mellitus (TD2), systemic arterial hypertension (SHA), obesity, or dyslipidemia]. SJL was calculated based on the absolute difference between mid-sleep time on weekends and weekdays. Current metabolic parameters and BP of the patients were compared with data from a year prior. Generalized estimating equations (GEE) and multiple linear regression analyses were used to examine the association among SJL, metabolic parameters, and BP. Multiple linear regression analyses adjusted for confounders showed that SJL was positively associated with the delta difference of fasting glucose (β = 0.11, *p* = 0.02) and triglyceride levels (β = 0.09, *p* = 0.04) among all subjects with NCCDs, and with fasting glucose (β = 0.30, *p* = 0.0001) and triglyceride levels (β = 0.22, *p* = 0.01) in the TD2 group. GEE analysis demonstrated an isolated effect of SJL on diastolic BP. High SJL impaired clinical and metabolic control in individuals with NCCDs, leading to a worse profile after a 1-year follow-up, particularly among type II diabetics.

## Introduction

In the third millennium, non-communicable chronic diseases (NCCDs) are sweeping the globe (Boutayeb and Boutayeb, 2005; Schmidt et al., 2011). The burden of NCCDs affects countries worldwide, but with a growing trend in developing countries (Schmidt et al., 2011). In Brazil, NCCDs have become a major health priority, and 72% of all deaths in the country are now attributable to these diseases, particularly cardiovascular disease and type 2 diabetes (TD2) (Low et al., 2015). Behaviors such as smoking, alcohol intake, sedentary lifestyle, and unhealthy diet are traditionally considered the main risk factors for NCCDs (Boutayeb and Boutayeb, 2005; Low et al., 2015). However, recent studies have identified disturbances in circadian rhythm, as well as sleep debt, as risk factors for obesity (Roenneberg et al., 2012; Parsons et al., 2015), diabetes, atherosclerotic cardiovascular disease (Wong et al., 2015), and metabolic syndrome (Parsons et al., 2015). Social jetlag serves as a proxy of these disturbances, measuring the discrepancy between circadian and social clocks based on the difference between sleep times on workdays and days off (Wittmann et al., 2006). This discrepancy is associated with internal desynchrony between the phase of the central brain clock in the suprachiasmatic nuclei and peripheral clocks (Potter et al., 2016; Sudy et al., 2019), potentially contributing to the development of NCCDs.

In a previous cross-sectional study, we examined the associations of social jetlag with obesity status and metabolic parameters among individuals with NCCDs. Information regarding BMI and biomarkers for metabolic syndrome was used to classify the participants as obese/non-obese and metabolically healthy/unhealthy. Participants with social jetlag > 1 h had a significant odds ratio for being overweight/obese and metabolically unhealthy (Mota et al., 2017). In order to further elucidate the mechanisms underlying this result, we conducted a subsequent study exploring food consumption and late meal timing in individuals with NCCDs and their relationship with social jetlag. Our findings showed that participants with social jetlag > 1 h had a higher intake of calories, protein, total fat, saturated fat, cholesterol, and larger servings of meat, eggs, and sweets than individuals with social jetlag ≤ 1 h. The authors also reported longer eating duration and later meal timing for breakfast, early afternoon snack and dinner (Mota et al., 2019).

Despite these indications of an influence of SJL on food consumption in other studies (Almoosawi et al., 2018; Cetiner et al., 2021), as well as on weight control (Roenneberg et al., 2012; Parsons et al., 2015; Zeron-Rugerio et al., 2019; Carvalho et al., 2021; Cetiner et al., 2021) and metabolic parameters (Wong et al., 2015; Espitia-Bautista et al., 2017; Koopman et al., 2017; Mota et al., 2017), no longitudinal follow-up studies of the relationship between SJL and metabolic control in patients with NCCDs have been conducted. Therefore, results described in the literature generally refer to a single timepoint (Wittmann et al., 2006; Wong et al., 2015), while the influence of SJL on the variation in metabolic parameters over time has not been investigated. Thus, this retrospective, longitudinal study investigated the influence of SJL on metabolic parameters and BP in patients with NCCDs who had regular clinical follow-up over a 1-year period. We hypothesize that SJL is negatively associated with metabolic control and positively associated with BP among individuals with NCCDs.

## Materials and Methods

### Subjects and Ethics

This retrospective, longitudinal study involved volunteers with NCCDs attended at outpatient clinics of the public health service of Uberlândia city, Minas Gerais State, Brazil, and was approved by the Ethics Committee of the Federal University of Uberlândia (permit no. 005464/2015). All volunteers signed a written informed consent form to participate in the study.

The public health service provides outpatient health care to patients with NCCDs, regular monitoring of metabolic parameters, and medicines to control these diseases. To be eligible to take part in the study, individuals had to have a confirmed pre-diagnosis of at least one of the following chronic diseases: obesity, systemic arterial hypertension (SHA), TD2, or dyslipidemia (hypercholesterolemia, hypertriglyceridemia, or low HDL-C). A total of 792 patients were invited to take part, of which 625 met the eligibility criteria and were subsequently included in the study. Individuals were excluded from the study if time since diagnosis of chronic diseases was less than 1 year (*n* = 2), data for assessments at two time points required for the study were unavailable (Garaulet et al., 2013), they were < 20 years of age or > 80 years of age (*n* = 7), pregnant (*n* = 1), shift workers (*n* = 2) or ex-shift worker (*n* = 29), had diseases or complications such as renal failure (*n* = 1), angina pectoris (*n* = 3), heart disease (*n* = 8), history of heart attack (*n* = 7), fasting glucose > 300 mg/dL (*n* = 9) or were an insulin user (*n* = 45).

**FIGURE 1:**
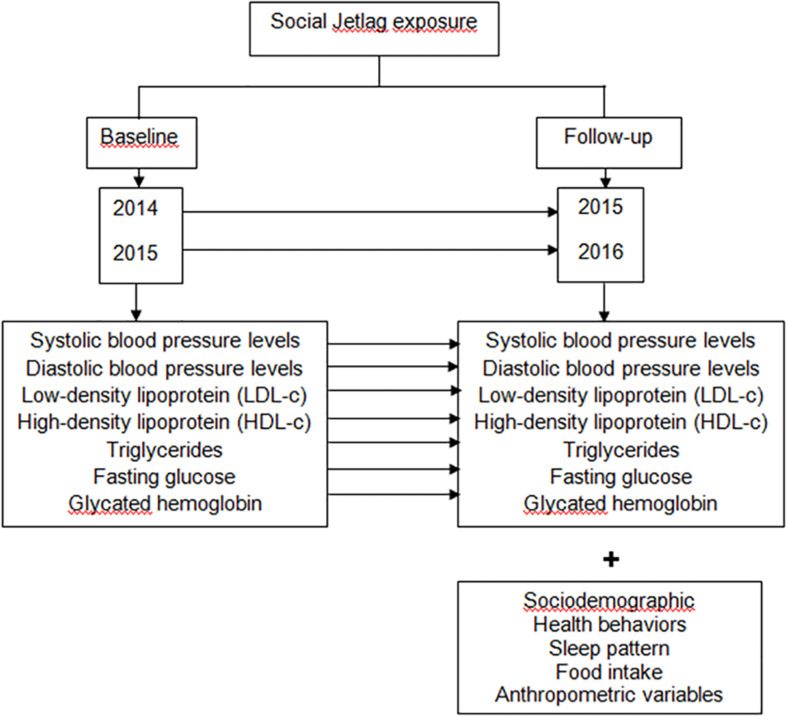
Methods to examine the influence of SJL on metabolic parameters and blood pressure (BP) over a year of follow-up. In this retrospective and longitudinal study the degree of exposure to SJL was assessed at the time of follow-up and the metabolic parameters were compared in two different times. Our assessments were carried out from September 2015 to July 2016; and in the present study we included only those patients who had in the medical record metabolic parameters referring to the year prior to the interview and anthropometric evaluation. Thus, the evaluation of the metabolic parameters was as follows: for the individuals interviewed in 2015, the metabolic parameters for the year 2014 were retrieved from the electronic medical record, with a 12-month interval; and for individuals interviewed in 2016, the metabolic parameters for 2015 were retrieved from the electronic medical record, with a 12-month interval. SJL, social jetlag.

All volunteers answered a questionnaire collecting demographic data for variables including age, sex, years of education, marital status, family income, and work status. Participants were also asked about health behaviors related to physical activity, alcohol intake, smoking, and the use of medicines. The methods used in this study for evaluating sleep patterns, clinical, metabolic and anthropometric aspects, food intake and physical activity have been described elsewhere (Mota et al., 2017). These assessments were carried out between September 2015 and July 2016. The metabolic parameters for the previous year were retrieved from patient electronic medical records with a 12-month interval. Information detailing the study design is presented in Figure 1.

### Sleep Patterns

Participants were asked to report usual bedtime, wake-up time, sleep-onset latency and usual sleep duration on weekdays and weekends. The questions used in the survey were: “What time have you been going to sleep on weekdays during the past two weeks?”; “What time have you been waking up on weekdays during the past 2 weeks?”; “What time have you been going to sleep on weekends during the past 2 weeks?”; “What time have you been waking up on weekends during the past 2 weeks?”; “How many minutes, on average, do you stay awake in bed before falling asleep after lights out?” Sleep duration was computed using the weighted average of self-reported sleep duration taking into consideration both weekdays and weekends: [(Reported current weekday sleep duration × 5) + (Reported current weekend sleep duration × 2)]/7 (Reutrakul et al., 2013). The volunteer was also asked about maintaining this sleep routine over the past year.

### Social Jetlag and Chronotype

Social jetlag was calculated based on the absolute difference between mid-sleep time on weekends (MSF) and weekdays (MSW) and was categorized dichotomously as SJL > 1 h or <1 h (Wittmann et al., 2006). Chronotype was derived from MSF time, with a further correction for sleep debt (MSFsc) calculated as the difference between average sleep duration on the weekend and average sleep duration during the week (Roenneberg et al., 2012).

### Anthropometric Variables

Anthropometric measurements were obtained by an expert team. Weight was measured on a set of scales accurate to the nearest of 0.1 kg (Welmy^®^) and height was measured using a wall-mounted stadiometer accurate to the nearest 0.1 cm (Welmy^®^). Body mass index (BMI, kg/m^2^) was calculated with BMI values >25 kg/m^2^ and BMI >30 kg/m^2^ classified as overweight or obese, respectively (WHO, 2000). Waist circumference (WC) was measured adopting the standard proposed by the WHO, 2000, where WC values ≥ 102 cm for men and ≥ 88 cm for women were considered high (WHO, 2008).

### Metabolic Parameters and Blood Pressure

Clinical and biochemical information collected included systolic and diastolic BP levels, lipid profile (LDL-c; HDL-c and triglycerides), and glucose profile (fasting glucose and glycated hemoglobin (HbA1c). The electronic medical system holds detailed data on the monitoring of the metabolic parameters and also on the clinical characteristics of the patients attended by the public health system. Only those individuals who had, according to their electronic medical record, two measures of BP, and glycemic or lipid profiles during the past year were included in the study.

All volunteers were instructed to follow a period of fasting for 12 h and, at the time of collection were asked whether this period had been adhered to. The collection, processing and analysis of blood samples for the determination of biochemical markers were performed on weekdays in the morning period. Analyses of these parameters were performed at a single laboratory used in the public health service and which follows widely established consolidated standards. BP measurement was performed by a doctor or nurse on weekdays, following all widely established protocols to determine blood pressure levels (Grupo de Trabalho Mapa and Grupo de Trabalho Mrpa, 2011).

### Food Intake

Dietary intake was assessed by a single 24-h food recall (24 h-FR), a methodology previously used in other studies in the nutrition area (Zamora-Ros et al., 2012; Ford et al., 2013; Murphy et al., 2013; Briefel et al., 2015). The volunteers discussed their reported food intake with a qualified nutritionist, and the information was amended to include additional explanations and details. The volunteers were instructed to provide as much detail as possible on meal times and food and fluids consumed on the day before the interview, including brand names and recipes for home-cooked foods. In this study, total intakes of energy and calories after 21:00 h. were employed as adjustment variables for the analyses performed (Tables 2, 3, respectively). The analysis of energy and nutrient intake was performed using the Virtual Nutri Plus software^®^.

### Statistical Analysis

All statistical analyses were performed using SPSS version 20.0 (SPSS Inc., Chicago, IL, United States) software, and *p* < 0.05 was considered statistically significant. Initially, normality of the data was tested using the Kolmogorov–Smirnov test. Categorical variables were summarized as frequencies and percentages, whereas continuous variables were expressed as mean and standard deviations or median and interquartile ranges. To characterize participants according to the degree of SJL, Pearson’s chi-square test was used to compare proportion variables, while Student’s *t*-tests or Mann–Whitney test for independent samples were used in comparisons for continuous variables.

Generalized estimating equations (GEE) were used to examine the effect of time (baseline and 1-year follow-up), SJL (categorized into ≤ 1 h or > 1 h) and its interaction with the metabolic parameters and BP, adjusting for confounders. GEE are an extension of generalized linear models and allow adjusting for correlations between observations. Robustness of the analyses derives from the fact that correct specification of the multivariate distribution is not required, only the average structure (Ziegler and Vens, 2010). The Sequential Sidak adjustment method was used for multiple comparisons. Effect size was determined by calculating Cohen’s d statistic and defined as small (*d* = 0.20), medium (*d* = 0.50) or large (*d* = 0.80) (Faul et al., 2007).

For determination of whether linear measure of SJL was associated with changes in metabolic parameters and BP, linear regression was performed, controlling for confounding factors. The difference between the two measurements (current measurement minus previous year’s measurement) for each parameter investigated was considered a dependent variable in the respective linear regression models. Variables that correlated for these differences with Pearson correlation or Spearman correlation (*r* > 0.20) were subjected to stepwise multivariate linear regression. SJL was treated as a continuous variable in all linear regression analyses. To remove the influence of multicollinearity from the multiple regression model, tolerance and variance-inflation factors (VIFs) were determined, and variables displaying tolerance < 0.1 or VIF > 10.0 were removed from the model.

Volunteers were divided into two groups: patients with TD2 (TD2 group) and patients without TD2 [SHA and/or obesity and/or dyslipidemia (SOD group)]. Thus, one or more of the NCCDs monitored in this study could be present in either of the groups. This division was performed because some results are important for certain conditions and show greater variability within groups, as is the case for blood glucose and HbA1c levels for people with diabetes. This division was applied for the linear regression analyses.

The power of the sample was calculated *a posteriori* using G^∗^Power software, with the mean difference test as a reference, total number of participants (625), 1 predictor (social jetlag), and an effect size of 0.02. The statistical power of the sample was 0.94.

## Results

### Participant Characteristics According to SJL ≤ 1 h or > 1 h

Participants’ sociodemographic characteristics, work factors, health behaviors, chronic diseases, and anthropometric and circadian variables according to SJL (≤ 1 h or > 1 h) are shown in Table 1. This study included 625 individuals. Most participants were female (*n* = 476; 76%), married (*n* = 315; 50.5%), and had < 12 years of education (*n* = 413; 66%).

**TABLE 1:** Demographic characteristics, work factors, health behaviors, chronic diseases, and anthropometric and circadian variables, according to social jetlag (*n* = 625).

Variables	All (*n* = 625)	SJL ≤ 1 h (*n* = 470)	SJL > 1 h (*n* = 155)	p*
Age (years)	56.0 + 12.0	58.2 ± 11.2	49.5 ± 12.2	**< 0.001**
Female (%)	476 (76.0)	353 (75.1)	123 (79.4)	0.19
Marital status–Married (%)	315 (50.5)	237 (50.5)	78 (51.3)	0.63
Family income–(US $553.00)	400 (64.1)	302 (64.4)	98 (63.2)	0.35
Education–≤ 12 years	413 (66.0)	320 (65.3)	93 (56.0)	0.15
**Work factors**				
Employment status–Employed (%)	346 (55.4)	236 (50.2)	110 (70,9)	**< 0.001**
Retired (%)	254 (40.6)	217 (46.1)	37 (23.8)	
Others (%)^∞^	25 (4.0)	18 (3.7)	7 (4.5)	
Hours worked per week	40.2 + 9.0	40.4 + 9.6	39.6 + 7.2	0.53
**Health behaviors**				
Smoking status–Yes (%)	84 (13.1)	57 (12.1)	25 (16.1)	0.20
Alcohol intake–Yes (%)	176 (28.2)	122 (26.0)	54 (34.0)	0.09
Alcohol–Servings/week	2.0 (0.5–6.0)	2.0 (0.5–6.0)	3.0 (1.8–8.5)	**0.01**
Physical activity (PA)–Yes (%)	231 (37.0)	181 (38.5)	50 (32.3)	0.55
Use of hypertensive drugs	471 (67.0)	364 (68.8)	107 (61.5)	0.07
Minutes of PA/week	180 (120–300)	180 (120–320)	180 (100–300)	0.41
**Anthropometry**				
BMI (kg/m^2^)	29.5 + 5.6	30.4 ± 5.7	30.4 ± 5.7	**0.01**
Overweight (BMI > 25 kg/m^2^)	454 (72.6)	329 (70.2)	125 (80.6)	**0.01**
Obese (BMI > 30 kg/m^2^)	332 (53.1)	257 (52.8)	88 (54.2)	0.75
Abdominal obesity (%)^§^	545 (87.2)	407 (86.6)	138 (89.0)	0.64
**Circadian**				
Chronotype–MSFsc (h)^†‡^	02:50 (02:00–03:40)	02:36 (01:09–03:41)	03:55 (02:45–04:42)	**< 0.001**
Bedtime weekday (h) ^‡^	22:15 (21:20–23:15)	22:20 (21:25–23:40)	22:50 (21:30–23:40)	**0.01**
Bedtime weekend (h) ^‡^	22:50 (21:50–23:50)	22:30 (21:30–23:30)	23:20 (22:20–00:40)	**< 0.001**
Wake time weekday (h) ^‡^	06:00 (05:30–07:00)	06:00 (06:00–07:00)	06:00 (05:30–07:00)	0.33
Wake time weekend (h) ^‡^	07:00 (06:00–08:30)	06:30 (06:00–08:00)	09:00 (08:00–10:00)	**< 0.001**
Sleep duration weekday (h)	8.0 (6.5–9.0)	7.5 (6.5–9.0)	8.0 (7.0–9.4)	0.22
Sleep duration weekend (h)	8.0 (7.0–9.5)	8.0 (7.0–9.0)	9.0 (8.0–10.0)	**< 0.001**
Mean sleep duration (h)	7.3 + 1.6	7.2 + 1.3	7.6 + 1.6	**0.01**

*Values expressed as mean and standard deviations for normally distributed data or as median (interquartile range) for non-normally distributed data. Pearson’s chi-square test was used to compare proportion variables, while Student’s *t*-tests or Mann–Whitney test for independent samples were used in comparisons for continuous variables. Values in bold are statistically significant at *p* < 0.05. Time is presented in the 24-h clock format. ^†^Chronotype (MSFsc) was derived from time of mid-sleep on days off (weekend), with further correction for calculated sleep debt — the difference between mean sleep duration on weekends and weekdays. ^‡^Time is presented in the 24-h clock format. ^§^Waist circumference ≥ 102 cm for men and ≥ 88 cm for women defined abdominal obesity. BMI, body mass index; SJL, social jetlag. ^∞^Unemployed and housewives.

**TABLE 2:** Estimated measurements of metabolic parameters according to social jetlag (*n* = 625).

	Mean + SE				Effects					
	SJL ≤ 1 h (*n* = 470)		SJL > 1 h (*n* = 155)		Time		SJL		Time*SJL	
All (*n* = 654)	Baseline	1-year follow-up	Baseline	1-year follow-up	Df	p*	Df	p*	Df	P
Fasting glucose^†^, mg/Dl	105.5 ± 1.8	105.5 ± 2.5	109.2 ± 4.3	108.6 ± 4.2	1	0.89	1	0.42	1	0.89
HbA1c^†^,%	6.8 ± 0.1	6.6 ± 0.2	6.8 ± 0.1	6.8 ± 0.3	1	0.64	1	0.76	1	0.53
Total cholesterol^‡^, mg/dL	198.1 ± 2.6	200.5 ± 2.6	195.0 ± 3.9	203.0 ± 4.3	1	**0.02^a^**	1	0.93	1	0.20
HDL-c^‡^, mg/Dl	47.9 ± 0.7	48.3 ± 0.9	47.0 ± 1.3	47.9 ± 1.5	1	0.25	1	0.68	1	0.59
LDL-c^‡^, mg/Dl	119.3 ± 2.3	122.3 ± 2.3	116.9 ± 3.4	125.8 ± 3.8	1	**0.002^b^**	1	0.94	1	0.08
Triglycerides^‡^, mg/Dl	159.5 ± 5.0	162.4 ± 5.2	160.8 ± 8.9	153.1 ± 7.0	1	0.56	1	0.63	1	0.21
Systolic BP^§^, mm Hg	94.2 ± 1.2	95.6 ± 1.4	94.4 ± 2.1	95.1 ± 2.1	1	0.35	1	0.93	1	0.77
Diastolic BP^§^, mm Hg	115.6 ± 1.5	117.4 ± 1.5	119.0 ± 2.3	126.5 ± 3.1	1	**0.001^c^**	1	**0.03^d^**	1	0.07

*p-values calculated by generalized estimating equation (GEE). *p* < 0.05 was considered significant. ^†^Adjusted for age, sex, time since diagnosis of type 2 diabetes, minutes of physical activity per week, BMI, menopause status and total calorie intake. ^‡^Adjusted for age, sex, time since diagnosis of dyslipidemia, minutes of physical activity per week, BMI, menopause status and total calorie intake. ^§^Adjusted for age, sex, time since diagnosis of systemic arterial hypertension, minutes of physical activity per week, BMI, menopause status and total calorie intake. ^*a*^Isolated effect of time for total cholesterol. Baseline = 196.0 ± 2.2; 1-year follow up = 201.5 ± 2.4, *p* = 0.02; effect sizes (Cohen’s d) = 0.002. ^*b*^Isolated effect of time for LDL-c. Baseline = 117.8 ± 2.1; 1-year follow up = 123.8 ± 2.2, *p* = 0.002; effect sizes (Cohen’s d) = 0.002. ^*c*^Isolated effect of time for diastolic BP. Baseline (117.3 ± 1.3); 1-year follow up (121.6 ± 1.7, *p* = 0.001); effect sizes (Cohen’s d) = 0.003. ^*d*^Isolated effect of SJL for diastolic BP. SJL **≤** 1 h = 116.2 ± 1.4; SJL > 1 h = 122.5 ± 2.4, *p* = 0.03), effect sizes (Cohen’s d) = 0.006. Df, degree of freedom; SJL, social jetlag; HbA1c, glycated hemoglobin; SE, standard error. Values in bold are statistically significant.

**TABLE 3:** Associations of social jetlag with delta difference of metabolic parameters and BP over 1-year follow-up (*n* = 625).

	All (*n* = 625)		Type 2 diabetes (*n* = 178)		SOD (*n* = 447)	
	β	*p*	β	*p*	β	*P*
Fasting glucose, mg/dL	0.11(0.2−2.4)	**0.02^a†^**	0.30(2.5−9.2)	**< 0.001^b^** ^**€**^	−0.06(−1.2−0.3)	0.27
HbA1c,%	−0.02(−0.2−0.2)	0.80	−0.02(−0.3−0.3)	0.85	−0.02(−0.2−0.2)	0.86
Total cholesterol, mg/dL	−0.03(−0.9−4.6)	0.53	−0.08(−1.5−1.4)	0.93	−0.04(−1.0−0.5)	0.52
HDL-c, mg/dL	−0.08(−0.1−0.2)	0.10	−0.02(0.4−0.3)	0.80	−0.11(−1.0−0.02)	0.06
LDL-c, mg/dL	−0.07(−2.6−0.4)	0.15	−0.08(−3.8−1.5)	0.39	−0.06(−2.8−0.85)	0.62
Triglycerides, mg/dL	0.09(0.1−5.9)	**0.04^c^** ^‡^	0.22(1.6−14.4)	**0.01^d^** ^**€**^	0.03(−2.3−4.3)	0.56
Systolic BP, mm Hg	−0.01(−0.2−0.2)	0.74	−0.09(−3.3−0.9)	0.83	−0.09(−1.8−0.1)	0.10
Diastolic BP, mm Hg	−0.06(−0.07−0.2)	0.27	−0.14(−2.6−0.1)	0.08	−0.03(−1.0−0.5)	0.47

*Variables correlated on Pearson or Spearman correlation (*r* > 0.20) were subjected to stepwise multivariate linear regression. ^*a*^Multivariate linear regression analysis adjusted for age, sex, BMI, menopause status, time since diagnosis of type 2 diabetes, use of sleeping pills and total calories consumed after 9:00 p.m. ^*b*^Multivariate linear regression analysis adjusted for age, sex, BMI, time since diagnosis of type 2 diabetes, menopause status and total calories consumed after 9:00 p.m. ^*c*^Multivariate linear regression analysis adjusted for age, sex, time since diagnosis of dyslipidemia, and total calories consumed after 9:00 p.m. ^*d*^Multivariate linear regression analysis adjusted for age, sex, BMI, time since diagnosis of dyslipidemia, menopause status and minutes of physical activity per week. ^†^*r*^2^ adjusted = 0.04. ^€^*r*^2^ adjusted = 0.07. ^‡^*r*^2^ adjusted = 0.02. SOD, systemic arterial hypertension and/or obesity and/or dyslipidemia group; HbA1c, glycated hemoglobin.*

A total of 155 (25%) individuals experienced SJL > 1 h, and this group had a lower mean age (*p* < 0.001), higher mean alcohol intake (servings/week) (*p* = 0.01), higher percentage of employed individuals (*p* < 0.001), higher mean BMI (*p* = 0.01), and greater prevalence of overweight (*p* = 0.01) compared with individuals who had SJL ≤ 1 h (Table 1). Regarding the circadian data, individuals with SJL > 1 h had higher MSFsc (*p* < 0.001), later sleeping times both during the week (*p* = 0.01) and on weekends (*p* < 0.001) and later waking times on weekends (*p* < 0.001) compared to individuals with SJL ≤ 1 h. Sleep duration on weekends (*p* < 0.001) and mean sleep duration (*p* = 0.01) were also higher among individuals with SJL > 1 h compared to those with SJL ≤ 1 h (Table 1).

In the TD2 group, the most prevalent NCCD combinations were TD2, SHA, dyslipidemia and obesity (25%; *n* = 44), TD2, SHA and obesity (18%; *n* = 32) and TD2, SHA and dyslipidemia (16%; *n* = 29). In the SOD group, the most prevalent NCCD combinations were SHA and obesity (23%; *n* = 105), SHA and dyslipidemia (21%; *n* = 97), and SHA, dyslipidemia and obesity (14%; *n* = 63, data not shown in table).

### Associations of SJL With Anthropometric and Metabolic Parameters and Blood Pressure

Table 2 shows the estimated marginal means for metabolic parameters and BP according to SJL ≤ 1 h or > 1 h. No effect of time, SJL, or interaction of time and SJL, was observed on fasting glucose, HbA1c, HDL-c, triglyceride, and systolic BP levels. An isolated effect of time showed that cholesterol levels (baseline = 196.0 ± 2.2; 1-year follow-up = 201.5 ± 2.4; *p* = 0.02); LDL-c levels (baseline = 117.8 ± 2.1; 1-year follow-up = 123.8 ± 2.2; *p* = 0.002) and diastolic BP (baseline = 117.3 ± 1.3; 1-year follow-up = 121.6 ± 1.7; *p* = 0.001) increased after 1 year. Furthermore, an isolated effect of SJL on diastolic BP (SJL < 1 h = 116.2 + 1.4; SJL > 1 h = 122.5 + 2.4, *p* = 0.03) was found. A small effect size was evident for the four significant associations found in the analysis (isolated effect of time on total cholesterol levels, Cohen’s *d* = 0.002; LDL-c, Cohen’s *d* = 0.003; diastolic BP, Cohen’s *d* = 0.003; and isolated effect of SJL on diastolic BP, Cohen’s *d* = 0.006). The results of the *post hoc* test in the different JLS groups (≤1 h and >1 h) are described in Supplementary Table 1.

Multiple linear regression analyses with SJL and the delta difference changes in metabolic parameters and BP are shown in Table 3. After adjustments for possible confounding variables, SJL was positively associated with the delta difference in fasting glucose levels among all subjects (β = 0.11, *p* = 0.02, *r*^2^ adjusted = 0.04) and in the TD2 group (β = 0.30, *p* = 0.001, *r*^2^ adjusted = 0.07). A positive association was found between SJL and the delta difference in triglyceride levels (after adjusting for confounders) among all subjects (β = 0.09, *p* = 0.04, *r*^2^ adjusted = 0.15) and in the TD2 group (β = 0.22, *p* = 0.01, *r*^2^ adjusted = 0.02).

## Discussion

This study evaluated the influence of SJL on metabolic parameters and BP among individuals with NCCDs over a 1-year follow-up. We found that SJL was positively associated with the delta difference of fasting glucose and triglyceride levels in the overall sample and for the TD2 group. These associations persisted after adjustment for traditional factors involved in metabolic control, such as sociodemographic characteristics and health behaviors. These data corroborate our previous findings (Mota et al., 2017, 2019) and confirm our initial hypothesis that circadian misalignment, as measured by SJL, is negatively associated with the control of metabolic parameters among individuals with NCCDs.

Clinical studies in healthy individuals have shown that circadian misalignment can result in systematic increases in postprandial glucose and insulin levels (Scheer et al., 2009; Buxton et al., 2012) and a decrease in insulin sensitivity (Buxton et al., 2012; Leproult et al., 2014). In this regard, Wong et al. (2015), in a sample of middle-aged individuals (*n* = 490; age = 42.7 +7.4 years) showed SJL was positively associated with fasting insulin (β = 0.11, *p* < 0.05) and insulin resistance (β = 0.11, *p* < 0.05), but not with fasting glucose. In the present study, no evaluation of postprandial glucose or insulin levels was carried out, but, in contrast to Wong et al. (2015), a positive association was found between SJL and the delta difference of baseline and 1-year follow-up fasting glucose data (Table 3). The GEE analysis failed to show a significant effect of SJL or of interaction between time and SJL categories (≤ 1 h or > 1 h) on glucose levels. However, the results of the linear regression analysis, which considered the absolute distribution of exposure to SJL of the population, suggested this variable raises fasting blood glucose and impairs blood glucose control. Further studies should be conducted to confirm these results.

Previous studies involving healthy individuals (Parsons et al., 2015) and patients with type I diabetes mellitus (Larcher et al., 2016) have found an association between SJL and HbA1c levels. However, in the present study (Tables 2, 3), and in other investigations (Reutrakul et al., 2013; Anothaisintawee et al., 2017), no association of SJL with HbA1c levels was evident. Nevertheless, a low prevalence of SJL (>1 h), as found in the present study (*n* = 164; 25%), may influence results regarding an association of this parameter with conditions of circadian misalignment (Anothaisintawee et al., 2017). In fact, associations between HBA1c levels and SJL have been found in studies where the prevalence of SJL was higher: 82% (*n* = 548/667) (Parsons et al., 2015) and 49% (39/80) (Larcher et al., 2016). These findings underscore the need for further studies involving different populations and periods investigating all of these parameters.

There is scant data in the literature to help explain the association between SJL and increase in blood glucose levels over the 1-year follow-up (Table 3). One of the mechanisms described that might justify our results is the secretion of melatonin at night, which may have been interrupted by exposure to light and/or circadian misalignment (Larcher et al., 2015). Melatonin secretion and sympathetic overactivity influence glucose metabolism, and sleep mid-points (used for establishing degree of SJL) have been shown to correlate significantly with dim light melatonin onset (Terman et al., 2001; Kantermann et al., 2015). In animals, reduced melatonin levels are associated with an increased risk of developing TD2 (Forrestel et al., 2017). In humans, lower melatonin secretion was associated with a significantly higher incidence of TD2, even after adjusting for confounding factors (McMullan et al., 2013). Among night workers, a group considered chronically exposed to circadian misalignment, melatonin production was negatively correlated with SJL (Vieira et al., 2021). Thus, studies have proposed that pancreatic receptors of β-cells for melatonin are coupled to three parallel signaling pathways with different influences on insulin secretion (Sharma et al., 2015). On the other hand, sympathetic overactivity and unfavorable changes in the hypothalamic–pituitary–adrenal axis lead to increased levels of catecholamines, cortisol, and appetite-satiety hormones (Depner et al., 2014; Rutters et al., 2014; Briancon-Marjollet et al., 2015; Larcher et al., 2015). These hormonal changes can promote the development of impaired glucose tolerance, insulin resistance, pancreatic β-cell dysfunction (Leproult et al., 2014), and an atherogenic lipid profile (Broussard et al., 2015; Chua et al., 2015).

The negative influence of SJL on glycemic and lipid parameters seen in the present study may also be explained by desynchronization between central and peripheral clocks (Sudy et al., 2019). This circadian misalignment is considered a stress factor (Wittmann et al., 2006; Puttonen et al., 2010) which, in turn, is associated with impairments in the metabolism of glucose and insulin (Seematter et al., 2005). Circadian misalignment can also impair lifestyle habits, such as engagement in physical activity (Alves et al., 2017) and dietary pattern (Silva et al., 2016; Zeron-Rugerio et al., 2019; Carvalho et al., 2021; Cetiner et al., 2021). In an animal study, SJL combined with a cafeteria diet led to overconsumption of food, increasing both bodyweight (16%) and triglycerides levels (Espitia-Bautista et al., 2017). As outlined earlier, our previous findings showed that SJL was associated with higher consumption of calories, protein, total fat, saturated fat, cholesterol, and larger servings of meat, eggs and sweets, as well as with late meal timing for breakfast and dinner (Mota et al., 2019). It is also important to consider a possible bidirectional relationship between SJL and lifestyle habits. While SJL may predispose to impairments in lifestyle habits, impairments in lifestyle may also increase SJL. Although the mechanisms explaining how lifestyle habits can increase circadian misalignment are not fully elucidated, it is known that genetics (Takahashi et al., 2018), humoral factors, eating habits and behavioral factors (Salgado-Delgado et al., 2010) contribute to the development of circadian misalignment. Additional studies are needed to better understand the causal relationship between lifestyle habits and SJL.

In the present study, the individuals with SJL > 1 h had higher mean BMI and greater prevalence of overweight (Table 1). These results mirror the findings of other studies on SJL (Roenneberg et al., 2012; Wong et al., 2015). Roenneberg et al. (2012) conducted an epidemiological study showing that SJL increased the probability of belonging to the group of overweight participants. Wong et al. (2015) found positive associations of SJL with BMI (β = 0.16, *p* = 0.001) and WC (β = 0.18, *p* < 0.001) in a population of healthy middle-aged adults (age 42.7 +7.4 years). The circadian system governs whole-body energy homeostasis and disruption in circadian clocks can lead to inadequate weight gain (Roenneberg et al., 2012; Garaulet and Gomez-Abellan, 2014). Factors related to food intake, such as changes in meal distribution throughout the day (Garaulet et al., 2013; Mota et al., 2019) and/or type of food consumed (Silva et al., 2016; Cetiner et al., 2021), and physical activity pattern (Rutters et al., 2014; Alves et al., 2017) may also explain weight change due to circadian disturbances. Other studies have failed to find associations between SJL and anthropometric parameters (Rutters et al., 2014; Alves et al., 2017; Skjakodegard et al., 2021). However, social jetlag was found to be associated with percentage weight loss, body weight loss (kg) and BMI reduction in bariatric patients 6 months after surgery (Carvalho et al., 2021). Taken together, these results point to the need for further studies investigating the effects of circadian misalignment on weight control.

Circadian misalignment may lead to an increase in BP (Scheer et al., 2009). Scheer et al. (2009) found, in a study under laboratory-controlled conditions, a 3% increase in mean arterial pressure during short-term circadian misalignment (*p* = 0.001). In contrast with previous reports (Rutters et al., 2014; Wong et al., 2015), however, no association between SJL and systemic BP values was found on linear regression analyses in the present study (Tables 2, 3). We found only an isolated effect of SJL on diastolic BP (Table 2). Despite the known relationship between stress and increased blood pressure due to increased activation of catecholamines and adrenergic stimulation (Paravati et al., 2021), also occurring in circadian misalignment conditions (Koch et al., 2017), the absence of other significant results for systolic blood pressure and the small effect size (Table 2) precludes the drawing of any meaningful conclusions. The patients included in the study should have controlled blood pressure values, given the regular monitoring of BP and use of hypotensive drugs prescribed in the outpatient follow-up. These factors can favor the control of BP (Lo et al., 2014). Future studies involving different population profiles and protocols should be conducted to confirm whether circadian misalignment leads to increased BP.

This study has some limitations. The generalization of the study results is relatively limited because users of the Brazilian public health service were included, comprising approximately 70% of the population. We used questionnaires that, although validated by other studies, are subjective and dependent on the memory and motivation of the participants. Also, we applied a single 24 h-FR, while both food intake and physical activity pattern were evaluated for a single timepoint, possibly constituting a poor representation of typical dietary pattern and usual physical activity. However, this data was only used as adjustment variables in the statistical analyses performed. In addition, studies in populations with similar characteristics to the present study sample have revealed there is little or no variation in feeding behavior (Oza-Frank et al., 2009; de Abreu et al., 2014) or physical activity level (Booth et al., 2017) over a 1-year follow-up. Lastly, we evaluated SJL retrospectively at a single timepoint and this assessment was based on sleep times during the previous 2 weeks, which may represent a limitation since the measure covers a relatively short period. Although volunteers reported maintaining their normal sleep habits, the current values of SJL may have changed during the course of the study period. However, several studies conducted in populations with similar sociodemographic characteristics and lifestyles to the group included in the present study have indicated that sleep pattern tends not to change significantly over a 1-year period (de Abreu et al., 2014; Lo et al., 2014; Song et al., 2016; Virta et al., 2013). Further studies exploring all these variables and incorporating alternative designs, such as longitudinal and prospective, may lead to a better understanding of the influence of circadian misalignment on control of metabolic parameters in individuals with different NCCDs.

The strengths of the present study were the use of a measure of circadian misalignment able to describe the chronic jetlag-like phenomenon induced by work or study schedules, reflecting a misalignment between the individual’s endogenous circadian clock and actual sleep times (Wittmann et al., 2006). This is the first study involving SJL and the metabolic control of NCCDs—conditions that are now highly prevalent and involve multiple risk factors (Boutayeb and Boutayeb, 2005; Schmidt et al., 2011; Low et al., 2015). Thus, social jetlag adds to the body’s risk factors for these diseases. The study results suggest that maintaining regular sleeping and waking times on weekdays and weekends—balancing social demands and biological preferences—may become an additional strategy in the multidisciplinary treatment of individuals with NCCDs, thus demonstrating the translational potential of evidence found in the present study.

This study presents the first evidence that SJL may adversely influence the control of metabolic markers related to NCCDs over time. The effects on fasting glucose and triglyceride levels observed are especially noteworthy. Our follow-up study of patients with NCCDs contributes to the emerging body of evidence supporting a link between circadian alignment and metabolic parameters, and corroborates the findings of our previous studies. The results also suggest that the adverse impact of SJL may be clinically significant for those with impaired glycemic and lipid profiles. Interventional studies are needed to determine the effectiveness of lifestyle modifications, such as correcting circadian misalignment.

## Data Availability Statement

The original contributions presented in the study are included in the article/Supplementary Material, further inquiries can be directed to the corresponding author/s.

## Ethics Statement

The studies involving human participants were reviewed and approved by Ethics Committee of the Federal University of Uberlândia (protocol no. 005464/2015). The patients/participants provided their written informed consent to participate in this study.

## Author Contributions

MM participated in the design, collection and analysis of data, and preparation and writing of the text. CS participated in the collection of data and the revision and approval of the manuscript. LB and WF participated in the collection of data and the final approval of the manuscript. CM and EM participated in the preparation and writing of the text and the final approval of the manuscript. CC participated in the design, writing, revision, and final approval of the manuscript. All authors contributed to the article and approved the submitted version.

## Conflict of Interest

The authors declare that the research was conducted in the absence of any commercial or financial relationships that could be construed as a potential conflict of interest.

## Publisher’s Note

All claims expressed in this article are solely those of the authors and do not necessarily represent those of their affiliated organizations, or those of the publisher, the editors and the reviewers. Any product that may be evaluated in this article, or claim that may be made by its manufacturer, is not guaranteed or endorsed by the publisher.
