# Supplementary material for: Social Jetlag Is Associated With Impaired Metabolic Control During a 1-Year Follow-Up
Source: Front Physiol. 2021 Sep 2;12:702769. doi: 10.3389/fphys.2021.702769 (PMC8445111; doi:10.3389/fphys.2021.702769)
Supplement: Supplementary file 1 [file Table_1.DOC]

**Supplement Table 1. Estimated measurements of metabolic parameters according to social jetlag** **(n=625).**

|  | **Mean + SE** | |  | **Mean + SE** | |  |
| --- | --- | --- | --- | --- | --- | --- |
|  | **SJL≤1h (n=470)** | |  | **SJL>1h (n=155)** | |  |
| **All (n=654)** | **Baseline** | **1-year follow-up** | **p*** | **Baseline** | **1-year follow-up** | **p*** |
| Fasting glucose^†^, mg/Dl | 105.5 + 1.8 | 105.5 + 2.5 | 0.98 | 109.2 + 4.3 | 108.6 + 4.2 | 0.98 |
| HbA1c^†^, % | 6.8 + 0.1 | 6.6 + 0.2 | 0.99 | 6.8 + 0.1 | 6.8 + 0.3 | 0.99 |
| Total cholesterol^‡^, mg/dL | 198.1 + 2.6 | 200.5 + 2.6 | 0.74 | 195.0 + 3.9 | 203.0 + 4.3 | 0.10 |
| HDL-c^‡^, mg/Dl | 47.9 + 0.7 | 48.3 + 0.9 | 0.93 | 47.0 + 1.3 | 47.9 + 1.5 | 0.63 |
| LDL-c^‡^, mg/Dl | 119.3 + 2.3 | 122.3 + 2.3 | 0.64 | 116.9 + 3.4 | 125.8 + 3.8 | **0.02**^‡^ |
| Triglycerides^‡^, mg/Dl | 159.5 + 5.0 | 162.4 + 5.2 | 0.84 | 160.8 + 8.9 | 153.1 + 7.0 | 0.96 |
| Systolic BP^§^, mm Hg | 94.2 + 1.2 | 95.6 + 1.4 | 0.63 | 94.4 + 2.1 | 95.1 + 2.1 | 0.98 |
| Diastolic BP^§^, mm Hg | 115.6 + 1.5 | 117.4 + 1.5 | 0.16 | 119.0 + 2.3 | 126.1 + 3.1 | **0.02**^§^ |

Notes: *p-values calculated by generalized estimating equation (GEE) (post-hoc test). p<0.05 was considered significant.. ^‡^Adjusted for age, sex, time since diagnosis of dyslipidemia, minutes of physical activity per week, BMI, menopause status and total calorie intake. ^§^Adjusted for age, sex, time since diagn­osis of systemic arterial hypertension, minutes of physical activity per week, BMI, menopause status and total calorie intake. SE: standard error.
